# Supplementary material for: High-dose intravenous immunoglobulins might modulate inflammation in COVID-19 patients
Source: Life Sci Alliance. 2021 Jul 28;4(9):e202001009. doi: 10.26508/lsa.202001009 (PMC8321664; doi:10.26508/lsa.202001009)
Supplement: Supplementary file 1 [file LSA-2020-01009_TableS1.pdf]

**Assay**	**Supplier**	**Kit**	**plasma dilution**	**biomarker**
Luminex	ThermoFisher Scientific	LHC6003M	1/2	FGF basic, IL1B, G-CSF, IL10, IL13, IL6, IL12(p40/p70), RANTES, EOTAXIN, IL17, MIP1α, GM-CSF, MIP1β, MCP1, IL15, EGF, IL5, HGF, VEGF, IFNγ, IFNα, IL1RA, TNFα, IL2, IL7, IP10, IL2R, MIG, IL4, IL8
ELISA	R&D Biosystem	DY126	1/2	Fas Ligand/TNFSF6
	R&D Biosystem	DY383	1/10000	sCD14
	R&D Biosystem	DY1607	1/500	sCD163
	R&D Biosystem	DY870-05	1/2000	LBP
	R&D Biosystem	DY156	1/2	B7-H1
	R&D Biosystem	DY375	1/3	TRAIL/TNFSF10
	R&D Biosystem	DY3078	1/2	FABP2
	R&D Biosystem	DY726	1/10	sTNF RII/TNFRSF1B
	R&D Biosystem	DY174	1/10	TACI / TNFRSF13B
	R&D Biosystem	DY2037	1/10	C5a
	Novus Biological	NBP2-66708	1/10	sC5b9
